# Psychosocial Work Environment, Stress Factors and Individual Characteristics among Nursing Staff in Psychiatric In-Patient Care

**DOI:** 10.3390/ijerph110101161

**Published:** 2014-01-20

**Authors:** Tuvesson Hanna, Eklund Mona

**Affiliations:** 1 School of Health Science, Blekinge Institute of Technology, SE-37971 Karlskrona, Sweden; 2 Faculty of Health and Society, Malmö University, SE-20506 Malmö, Sweden; 3 Department of Health Sciences, Lund University, P.O. Box 157, SE-22100 Lund, Sweden; E-Mail: mona.eklund@med.lu.se

**Keywords:** mental health care, nursing staff, psychosocial work environment, questionnaire, stress, troubled conscience

## Abstract

The psychosocial work environment is an important factor in psychiatric in-patient care, and knowing more of its correlates might open up new paths for future workplace interventions. Thus, the aims of the present study were to investigate perceptions of the psychosocial work environment among nursing staff in psychiatric in-patient care and how individual characteristics—Mastery, Moral Sensitivity, Perceived Stress, and Stress of Conscience—are related to different aspects of the psychosocial work environment. A total of 93 nursing staff members filled out five questionnaires: the QPSNordic 34+, Perceived Stress Scale, Stress of Conscience Questionnaire, Moral Sensitivity Questionnaire, and Mastery scale. Multivariate analysis showed that Perceived Stress was important for Organisational Climate perceptions. The Stress of Conscience subscale Internal Demands and Experience in current units were indicators of Role Clarity. The other Stress of Conscience subscale, External Demands and Restrictions, was related to Control at Work. Two types of stress, Perceived Stress and Stress of Conscience, were particularly important for the nursing staff’s perception of the psychosocial work environment. Efforts to prevent stress may also contribute to improvements in the psychosocial work environment.

## 1. Introduction

In many countries, there is great interest in the work environment and well-being of staff members in psychiatric care contexts [1,2,3,4,5,6,7]. The phenomenon of the psychosocial work environment and its constituents have been described in several different ways and often include a multitude of aspects, such as organisational climate and culture, work demands, work control, leadership empowerment and support, and co-worker support and collaboration [8,9,10]. Lindström *et al.* [9] described the psychosocial work environment as a compound system that includes the work, the workers, and the environment. It is a common assumption that the psychosocial work environment is important for the well-being of the staff [11]. For example, favourable work environments have been found to be related to work engagement in psychiatric in-patient care [12] and reducing the risk of burnout [13]. The opposite, a poor work environment, has been found to create stress and burnout [3,6,7]. Research characterising the work environment in psychiatric care has reported mixed findings. Among the negative sides are the psychiatric staff’s experiences of a demanding work environment with a heavy and intense work load [1,14] and the aggression and violence are commonly recognised problems [1,2,15,16]. The complex relationship with patients in psychiatric care has also been described as a source of stress [2,14,17]. Several studies have highlighted the beneficial aspects of working in psychiatric care, such as good morale [6,18], low levels of moral distress [19], and meaningful and stimulating work [14]. One study indicated that psychiatric nurses were satisfied with their work [20]. It is crucial to study possible antecedents that may contribute to the understanding of the psychosocial work environment of nursing staff (nurses and nurse assistants) in psychiatric in-patient care. Perceived Stress, Stress of Conscience, Moral Sensitivity, Mastery, and several individual characteristics are factors that have been shown to be associated with the psychosocial work environment, and they will be elaborated on later in this report. To the best of the authors’ knowledge, no previous study has addressed the relationships among these factors in the context of psychiatric in-patient care. A better understanding of such associations may open up new paths for creating ways to improve the psychosocial work environment of psychiatric nursing staff members and developing future work place interventions. Improving the nursing staff’s psychosocial work environment must be considered to be of vital importance.

One area that may be important to the psychosocial work environment is Perceived Stress, which can be understood to mean how unpredictable, uncontrollable, and overloaded a person perceives his/her life to be [21]. The literature in the area of stress in general is voluminous, and several studies from various countries have reported on moderate to high levels of stress, strain, and burnout among psychiatric nurses [3,4,5,20,22]. The impact of Perceived Stress on the psychosocial work environment in psychiatric care, however, has been scarcely researched and needs to be further investigated. Another type of stress worth considering is Stress of Conscience, which is a type of stress that can be described as “…a product of the frequency of the stressful situation and of the perceived degree of troubled conscience as rated by health care personnel themselves” (p. 636 in [23]). The construct of Stress of Conscience concerns the experiences of troubled conscience identified in interviews with health-care staff [24] and is thus empirically derived. Theoretically, a person’s conscience may be seen as an inner dialogue and guide that navigates people between right and wrong [25,26]. Studies on Stress of Conscience in psychiatric in-patient care are still rare. One previous study found that individual aspects such as moral burden and sense of mastery, as well as aggression and control at work, were related to Stress of Conscience [27]. Another study that included staff in psychiatric care found a relationship between burnout and Stress of Conscience [28]. Studies regarding Stress of Conscience in residential care, for example, have found that organisational and environmental support were associated with low levels of Stress of Conscience [29], and that noticing disturbing conflicts between co-workers and being exhausted were positively associated with Stress of Conscience [30]. Stress of Conscience has also been found to be related to burnout in elderly care [31]. Thus, Stress of Conscience may be a factor to consider in relation to nursing staff members’ perceptions of their psychosocial work environment.

Another aspect to consider in relation to the psychosocial work environment is Moral Sensitivity, which involves the staff’s attention to and awareness of moral conflicts, values, and implications. It also includes insight into the patient’s situation and has the function of a guide for ethical decision making and lays the ground for the ability to understand a patient’s needs [32]. Using principal component analysis, Lützén *et al*. [33] found three factors of Moral Sensitivity; Sense of Moral Burden, Sense of Moral Strength, and Moral Responsibility. A study on psychiatric care showed that nursing staff members with more experience had higher Moral Sensitivity compared with those with less experience [34]. In another study, one of the Moral Sensitivity factors, Moral Burden, was related to high levels of Stress of Conscience [27]. Studies performed in other contexts have found, for example, an association between Moral Sensitivity and psychosocial work environment among Iranian nurses [35], and the study by Park and colleagues [36] among nursing students in Korea showed that students at the end of the education process had higher Moral Sensitivity than those at the beginning of their education. An additional aspect that might be important in the understanding of the psychosocial work environment of nursing staff members is Mastery. Mastery can be understood as a coping mechanism, and it involves the control a person believes she/he has over situations and factors affecting her/his life. Pearlin and Schooler [37] defined Mastery as “…the extent to which one regards one’s life-chances as being under one’s own control in contrast to being fatalistically ruled” (p. 5). Few studies have examined Mastery among nursing staff in psychiatric in-patient care. In one study, Mastery worked as a protective factor against Stress of Conscience among psychiatric nursing staff [27]. The individual characteristics of Moral Sensitivity and Mastery could be factors that affect the way the nursing staff perceive the psychosocial work environment. Taking these two aspects into consideration may lead to further understanding of the psychosocial work environment in psychiatric in-patient care.

Several studies have indicated that individual characteristics might influence the way a person perceives the work situation. For example, registered nurses have been found to be more morally distressed than nursing assistants [19], and female psychiatric staff members have been found to experience more moral stress than men [34]. Furthermore, younger nurses had significantly higher emotional exhaustion scores in a Japanese study [5]. Studies that have compared nurses to nursing assistants in psychiatric care have yielded mixed results. Sørgaard *et al.* [7] found few differences between the two staff groups in terms of stress and burnout. There were also no differences between nurses and nursing assistants regarding perceived stress and Stress of Conscience in research performed by Tuvesson *et al.* [27]. In another study, the main stressors differed between nurses and nursing assistants. Nurses perceived a lack of resources to be the main stressors, while and nursing assistants perceived client-related difficulties to be the main stressors [38]. In order to make changes aimed at improving the psychosocial work environment, it might be important to clarify if and how nurses and nursing assistants differ in their perceptions of the work situation. It may also be important to explore how age, gender, and length of work experience are related to the psychosocial environment.

Nursing has basic common denominators regardless of context, but also distinct characteristics depending on the nature of the medical and health problems at hand, and such specifics tend to shape work. Mental illness may be life threatening and often results in added social complications, which makes nursing in inpatient psychiatry complex and challenging [18]. Organizational pressures in the complex psychiatric care system add further to high demands on the nursing staff [39]. They often have feelings of inadequacy, which has been shown to be related to Stress of Conscience [24,40]. In view of the findings presented herein, it is clear that there is a strong need for more research aimed at exploring the impact of stress factors and individual characteristics on the psychosocial work environment among nursing staff in psychiatric in-patient care. The aims of this study were to investigate perceptions of the psychosocial work environment among nursing staff members in psychiatric care and the potential importance of occupational group (nurses or nursing assistants), gender, age, and work experience in these perceptions. The aim was also to investigate how individual characteristics, Mastery, Moral Sensitivity, Perceived Stress, and Stress of Conscience are related to the psychosocial work environment.

## 2. Methods

This cross-sectional study was performed after approval by the Regional Ethics Review Board (Dnr 380/2008). Data was collected in 2009 at psychiatric in-patient wards.

### 2.1. Setting and Sample

The setting for this study was 12 psychiatric acute in-patient wards located in southern Sweden. The units admitted patients from the geographical areas for which they were responsible and had 13–16 beds, although all of the units were often overcharged. They were all acute general wards, admitting adult patients with mixed diagnoses. The admissions were both voluntary and coerced by law. The length of stay generally varied between two and five weeks. All nursing staff members (registered nurses and nursing assistants) who worked during the day and had worked at the unit for a minimum of two months were included in the study. Power analysis implied that a sample of 100 individuals was needed in order to achieve an effect size of 0.5, with 80% power at *p* ˂ 0.05 [41,42]. Questionnaires (see Section 2.3), to be completed anonymously, were given to 179 nursing staff members; 93 questionnaires were returned (from 38 nurses and 55 nursing assistants), which resulted in an overall response rate of 52.3%. A total of 78% of the participating nursing staff were women, and 86% were permanently employed at their units. Ages ranged from 21 to 65 years, with the nursing assistants being older (50 years) compared to the nurses (45 years) (Mann–Whitney *U*-test). The participants’ average length of work experience in psychiatric care was 18 years, and they had worked at their current units for on average of nine years. The nursing assistants had significantly longer average work experience in psychiatric care (20 years) than the nurses (15 years) (Mann–Whitney *U*-test). The non-responders did not differ from the participants regarding the investigated characteristics (*i.e.*, age, gender, proportion of nurses/nursing assistants, and length of work experience).

### 2.2. Data Collection

Clinical directors and unit managers gave their approval to conduct the study in the units. An information meeting was held at the 12 units, and questionnaires were distributed to the nursing staff attending the meeting. Nursing staff members who were not present at the information meeting received the questionnaires from the managers on their next working day. Together with the questionnaires, potential participants also received written information about the study and an informed consent form. The completed questionnaires and the signed informed consent forms were returned by post in sealed, prepaid envelopes. Three reminders were given to the wards over a period of four months.

### 2.3. Measurements

The QPSNordic 34+ was applied to measure psychosocial aspects of the work environment. The QPSNordic 34+ is a short version of the General Nordic Questionnaire for Psychological and Social Factors at Work (QPSNordic) [43,44,45] and consists of 37 items. Each item has five response alternatives, ranging from “very seldom or never” (1) to “very often or always” (5). In line with a previous study [46], subscales were identified by testing sets of items that corresponded to the subscales of the full version of the QPSNordic. The internal consistency of each of those sets of items was calculated, and a Cronbach’s alpha > 0.70 [47] was set as the criterion for keeping the scale for further analysis. Using these procedures, the following subscales were identified and found to have satisfactory internal consistency: Empowering Leadership (Cronbach’s alpha = 0.85), Role Clarity (Cronbach’s alpha = 0.79), Support from Superiors (Cronbach’s alpha = 0.80), Control at Work (Cronbach’s alpha = 0.72), and Organisational Climate (Cronbach’s alpha = 0.77).

General stress was measured by a 14-item scale named the *Perceived Stress Scale (PSS)* [21]. Each item has five response alternatives ranging from “never” (0) to “very often” (4). Higher scores indicate a high amount of Perceived Stress; the maximum score is 56. The psychometric properties of the scale have been well documented [21,48,49]. In the present study, the Cronbach’s alpha was 0.83.

Stress due to a troubled conscience was assessed using the *Stress of Conscience Questionnaire* (*SCQ*) [23]. This nine-item scale includes two parts for each item. Part A asks how often the participant has experienced a certain situation in the workplace, using a six-point Likert scale ranging from “never” (0) to “every day” (5). Part B consists of a 100-mm visual analogue scale (VAS) with the extreme points being 0 mm = “No, not at all” and 100 mm = “Yes, it gives me a very troubled conscience”. The scores from part A are multiplied with the scores from part B in order to create an index for each question. In this analysis, a total index comprising all nine items was used, as well as two aggregated scales (Internal Demands; External Demands and Restrictions), which have previously demonstrated good internal consistency [23].

The revised *Moral Sensitivity Questionnaire* (*MSQ*) was used to measure assumptions about Moral Sensitivity. It consists of nine items, and a score of 1 (“total disagreement”) to 6 (“total agreement”) is used. The MSQ is comprised of three subscales: Sense of Moral Burden, Sense of Moral Strength, and Moral Responsibility [33]. In the present study, the third subscale was analysed as two single items, due to poor internal consistency (Cronbach’s alpha = 0.34).

The *Mastery* scale is a self-report scale, developed to measure the participant’s feeling of having control over his/her life [37]. The scale consists of seven items that are rated according to a four-point response format, ranging from “strongly agree” (1) to “strongly disagree” (4). The sum of the seven items constitutes a total Mastery index. Satisfactory internal consistency was previously determined for the Swedish version [50], which was used in the present study.

The *nursing staff characteristics* included six questions regarding age, gender, occupational belonging, type of employment, and work experience.

### 2.4. Statistical Analysis

The material was not normally distributed; therefore, non-parametric statics were used. Descriptive analysis was undertaken for characterising the samples. The Mann–Whitney *U*-test was used to assess differences among subgroups of the sample. Spearman’s rank correlations were conducted to explore the relationships among variables and to identify which variables to include in subsequent logistic regression analysis. The variables that showed a relationship with the psychosocial work environment subscales at *p* ≤ 0.1 were entered as independent variables in a set of logistic regression models, with the psychosocial work environment factors as dependent variables. The logistic regression model used was a forward stepwise conditional model with dichotomous dependent and independent variables. The significance level was set at *p* < 0.05 and the software used was the Statistical Package for Social Sciences (SPSS), version 20 (IBM, Chicago, IL, USA).

## 3. Results

Table 1 summarises the mean values on the studied variables. The participants’ ratings of the psychosocial work environment varied among the subscales. A low level of Support from Superiors was indicated, but medium levels were indicated on the other subscales. The nursing staff members’ ratings of Stress of Conscience and Perceived Stress were predominately low, while Sense of Moral Burden was rated according to the middle alternatives of the scale. In addition, the respondents’ ratings of Moral Strength and Mastery were moderately high. There were no significant differences among groups based on any of the sample characteristics (age, sex, occupation, work experience) and the study variables (Mann-Whitney *U*-test).

Correlations among the psychosocial work environment variables and the selected covariates of Perceived Stress, Stress of Conscience, Mastery, Moral Sensitivity, and nursing staff characteristics are shown in Table 2. Empowering Leadership was positively correlated with Sense of Moral Strength and Mastery. The findings also indicated that several of the covariates were related to Role Clarity. Long experience in psychiatric care, in the current ward and high levels of Moral Strength were positively associated with a high rating of Role Clarity. Moreover, high levels of Perceived Stress, Stress of Conscience, and Sense of Moral Burden were negatively correlated with Role Clarity. With regard to Control at Work, there were significant negative associations with all of the Stress of Conscience variables. It further appeared that three of the covariates—Perceived Stress, External Demands and Restrictions, and Mastery—correlated significantly with Organisational Climate. None of the variables set as covariates showed any significant association with Support from Superiors.

**Table 1:** Mean values for the QPSNordic 34+, Stress of Conscience Questionnaire, Perceived Stress Scale, Moral Sensitivity Questionnaire, and Mastery scale.

Variables	Mean Score		
	Nurses (*n:* 38)	Nursing Assistants (*n*: 55)	Total Sample (*n*: 93)
**QPSNordic 34+**			
Empowering Leadership (2–10)	6.6	6.6	6.6
Role Clarity (2–10)	7.2	7.8	7.5
Control at Work (4–20)	12.4	11.6	11.9
Support from Superiors (2–10)	3.4	3.4	3.4
Organisational Climate (6–30)	18.4	18.4	18.4
Stress of Conscience (0–225)	43	40.1	41.4
Internal Demands (0–125)	24.7	24.5	24.6
External Demands and Restrictions (0–125)	27.6	25	26.1
**Perceived Stress (0–56)**	23.4	22.1	22.6
**Moral Sensitivity**			
Sense of Moral Burden (4–24)	13.6	14.2	13.9
Sense of Moral Strength (3–18)	14.2	15	14.7
**Mastery (7–28)**	22.7	22.8	22.8

The covariates that showed an association with the psychosocial work environment variables (*p* < 0.1) (Table 2) were entered in forward stepwise conditional logistic regressions. The results are presented in Table 3. Perceived Stress was of importance for Organisational Climate. Participants belonging to the high group regarding Perceived Stress had a four-fold higher risk of perceiving low levels on the Organisational Climate factor. The analysis regarding Role Clarity resulted in two significant factors: Internal Demands and Experience in the current ward. Belonging to the group that rated Internal Demands high increased the likelihood of perceiving a low level of Role Clarity by more than three times. The group with little experience working in the current ward had a four-fold higher risk of belonging to the low group on Role Clarity compared with those with longer experience. Finally, belonging to the high-level group of External Demands and Restrictions increased the risk by nearly three times of perceiving low levels of Control at Work.

**Table 2:** Correlations among psychosocial work environment (QPSNordic 34+), nursing staff characteristics variables, Perceived Stress Scale (PSS), Stress of Conscience (SCQ), Moral Sensitivity Questionnaire (MSQ), and Mastery scale.

Variables	*r*-scores				
	Empowering Leadership	Role Clarity	Control at Work	Support from Superiors	Organisational Climate
**Nursing staff characteristics**					
Age	−0.158	0.151	0.009	0.113	0.067
Experience in current ward	0.047	0.190 ^#^	0.062	0.085	0.148
Experience in psychiatry	−0.139	0.191 ^#^	−0.017	0.132	0.063
**PSS**	−0.172	−0.302 **	−0.136	−0.114	−0.265 *
**SCQ**					
Stress of Conscience	−0.086	−0.271 *	−0.282 **	−0.024	−0.153
Internal Demands	−0.063	−0.212 *	−0.215 *	−0.050	−0.044
External Demands and Restrictions	−0.126	−0.329 **	−0.337 **	−0.060	−0.234 *
**MSQ**					
Sense of Moral Burden	−0.175	−0.198 ^#^	−0.141	−0.135	−0.146
Sense of Moral Strength	0.194 ^#^	0.247 *	−0.124	0.069	0.109
Item nr 1	−0.051	−0.004	0.057	0.063	−0.125
Item nr 9	0.143	0.125	0.109	−0.093	0.067
**Mastery**	0.214 *	0.169	0.157	0.087	0.237 *

* *p* < 0.05; ** *p* < 0.01; ^#^ *p* < 0.1; Spearman’s rank correlation.

**Table 3:** Variables of importance to aspects of the psychosocial work environment.

Dependent variable	Independent variable	*p*	OR	95% CI for OR
Organisational Climate ^1^	Perceived Stress	0.003	4.71	1.722–12.907
Role Clarity ^2^	Experience in current ward	0.009	4.04	1.416–11.549
	Internal Demands	0.018	3.32	1.159–9.501
Control at Work ^3^	External Demands and Restrictions	0.009	2.93	1.205–7.099

Note: Analyses based on a forward stepwise conditional logistic regression (*p* ≤ 0.05). All three models exhibited acceptable goodness-of-fit (Hosmer–Lemeshow test, *p* > 0.05). ^1^ 15.7% explained variance (Nagelkerke *R*^2^); ^2^ 21.8% explained variance (Nagelkerke *R*^2^); ^3^ 8.9% explained variance (Nagelkerke *R*^2^).

## 4. Discussion

The present study set out to explore the psychosocial work environment among psychiatric nursing staff members in in-patient wards by investigating relationships among aspects of the psychosocial work environment and selected covariates of Perceived Stress, Stress of Conscience, Moral Sensitivity, Mastery, and nursing staff characteristics. Of the investigated psychosocial work environment variables, Role Clarity had the most significant associations with the covariates. According to the instrument used to assess aspects of the psychosocial work environment, Role Clarity was involved in how the work role was understood by the staff, whether the staff perceived they had clear goals for the work they were to perform, and whether the staff knew what was expected from them. The importance of perceiving role clarity has been illustrated in several studies. For example, a relationship between lack of role clarity and stress was found [51], and role conflict was positively associated with burnout among various health care personnel in a Hungarian study [52].

In the present study, high levels on the Stress of Conscience subscale Internal Demands was related to low Role Clarity. Internal Demands involves aspects of troubled conscience due to inner demands in terms of personal wishes and desires and ideal images. Troubled conscience is the result of being hindered from acting in accordance with one’s professional beliefs and doing what feels right [24]. In interview studies, Sorlie *et al.* [53,54] found that nurses experienced troubled conscience when there was a discrepancy between their images of care and the reality of care. Similar results were found in another study, wherein nurses perceived a conflict between the reality of in-patient care and their vision of psychiatric nursing [55]. These conflicting demands could be troublesome per se, and Juthberg *et al.* [26] suggested that the experience of a troubled conscience may be connected to the professional role. The present study indicates that Stress of Conscience, in terms of Internal Demands, may infer a lack of clarity regarding professional roles. Finding ways to prevent conflicting Internal Demands should therefore be of high interest. Measures for achieving this may include easy access to supervision and a culture that enables staff to express their feelings openly and handle frustration, as proposed in a literature review by Dickinson and Wright [56]. That review also indicated that continued professional development, such as psychosocial interventions training, would reduce stress and burnout. It seems reasonable to assume that knowledge generally strengthens people’s ability to handle stressful situations, also conflicting internal demands. Moreover, clinical supervision may be a core avenue to reflecting on moral and ethical concerns and thus reducing stress related to such conflicts among psychiatric nursing staff [56].

Strategies such as these could not only help to prevent Stress of Conscience but also reduce the risk of lack of Role Clarity. Ward managers could seek to create opportunities for the nursing staff to maintain their professional beliefs, and in so doing, prevent Stress of Conscience and strengthen the professional roles of the nursing staff.

The logistic regression analyses also showed that the nursing staff’s work experience in the current ward was an important factor in explaining variation in Role Clarity. A few studies have previously found length of work experience to be important in relation to different aspects of the professional roles of psychiatric staff. For example, a positive relationship between the length of nurses’ experience in psychiatric care and role competency has been found [57], and newly qualified psychiatric nurses have been found to perceive their roles as unclear [58]. These findings point to a need for developing clearer professional roles in psychiatric care that can be adopted easily by new staff. Several studies have illustrated the complex reality of psychiatric nursing and the professional roles of staff in psychiatric care. Questions have been raised and discussed regarding what it is that constitutes psychiatric nursing and the identity of the psychiatric nurse [59]. The wide range of tasks performed by psychiatric nurses has been described as one reason for the lack of clarity of roles in psychiatric care. Another reason is the gap between theory and practice [58]. One study revealed that nurses in psychiatric care perceived their work description as unclear, and that they were confused about what their main tasks were [60]. Education can play a vital role in the reconceptualization of psychiatric nursing and the preparation of students for their future work [61]. Educational staff and psychiatric ward managers would thus be able to prevent lack of Role Clarity by establishing clear descriptions of what psychiatric nursing is and the main goals of psychiatric nursing on the unit.

The findings of the present study also highlight the importance of Perceived Stress for the nursing staff’s perceptions of the Organisational Climate. High ratings of Perceived Stress were related to low scores on Organisational Climate. Organisational Climate assesses perceived levels of encouragement, support, rewards, relaxing and comforting climate, and communication within the organisation and among co-workers. Few studies seem to have addressed this relationship in psychiatric in-patient care, but a study that included psychiatric nurses found a significant correlation between Organisational Climate and job satisfaction [62]. In other health care contexts, studies have found, for example, that Organisational Climate was associated with empowerment [63], nursing competency [64], and nurses’ intent to stay at work [65]. One aspect of the Organisational Climate concerns perceived support. Previous studies in psychiatric care have found that high levels of support from co-workers were related to lower levels of emotional exhaustion [38], and that social support was important in relation to stress and burnout among Jordanian psychiatric nurses [17,20]. The findings of the present study highlight the reverse relationship between Perceived Stress and perception of the Organisational Climate. Efforts to reduce the risk of stress are always important and may be further motivated by the fact that it may be beneficial in reducing nursing staff members’ apprehensions regarding the Organisational Climate.

The findings further suggest that high levels on the Stress of Conscience subscale External Demands and Restrictions were related to a low sense of Control at Work. The External Demands and Restrictions factor addresses outer circumstances that influence a person’s work and lead to a troubled conscience [24]. Several studies have indicated that work control is important for the well-being of the staff in psychiatric care contexts. A low level of control has been found to be associated with the feeling of being uptight and tense [5], emotional exhaustion [66], and poor psychological health [67]. One study has also indicated that psychiatric staff members experience a loss of power due to constant changes and improvements initiated by the managers [14]. It is possible that outer circumstances, such as constant changes in the workplace, may lead to contradicting External Demands and Restrictions and a troubled conscience, which in turn affect the nursing staff’s sense of Control at Work. One may also speculate that nursing staff members may be hindered from acting in accordance with their beliefs due to external factors at work, and that this may lead to a sense of loss of control in the workplace.

Results from the present study offer an initial step towards exploring the relationships between the psychosocial work environment and aspects of stress, Moral Sensitivity, Mastery, and individual characteristics among nursing staff members in psychiatric in-patient care. However, generalisability of these results is limited, due to a relatively small sample size. The response rate was not ideal (52.3%), but the sample in the present study seems to be representative of the staff at the 12 included wards in terms of age and proportion of nurses to nursing assistants. The transferability to other settings, however, must be regarded as limited. The subscale Moral Responsibility demonstrated poor internal consistency, and was therefore analysed as two single items. Despite these limitations, however, the present study contributes some understanding of the psychosocial work environment in psychiatric in-patient care.

## 5. Conclusions

The findings of the present study highlight the importance of stress, in terms of Perceived Stress and Stress of Conscience, in the nursing staff’s perceptions of the psychosocial work environment. In a two-pronged attempt to plan workplace interventions, it might be possible to focus on preventing stress at the same time as enhancing the psychosocial environment in terms of Organisational Climate, Role Clarity, and Work Control. Furthermore, by organising the work in such a way that a troubled conscience is counteracted, Role Clarity and Work Control may be strengthened. It is an important responsibility of managers of psychiatric in-patient units to create a climate and opportunities that enable the nursing staff to discuss and reflect on moral and ethical concerns, in order to maintain their professional and moral beliefs. Another conclusion is that newly employed nursing staff members may lack a sense of Role Clarity on the current unit. By communicating and establishing what psychiatric nursing is about, including its goals, it might be possible to strengthen the professional role among inexperienced nursing staff members. Clinical supervision might be essential in counteracting both stress and indistinct work roles. These conclusions may be seen as hypotheses for future research, which is needed to better understand the potential profits of creating interventions aimed at reducing stress and troubled consciences.
